# No congruency sequence effect across Simon and Eriksen tasks with aligned temporal processing dynamics: Evidence for domain-specific over domain-general cognitive control

**DOI:** 10.3758/s13421-025-01758-1

**Published:** 2025-07-21

**Authors:** Daniel Bratzke, Ruben Ellinghaus, Ian Mackenzie, Victor Mittelstädt

**Affiliations:** 1 Department of Psychology, University of Bremen, Bremen, Germany; 2 Department of General Psychology: Judgment, Decision Making, Action, Faculty of Psychology, University of Hagen (FernUniversität in Hagen), Hagen, Germany; 3 Department of Psychology, Eberhard Karls University of Tübingen, Tübingen, Germany

**Keywords:** Cognitive control, Selective attention, Congruency sequence effect

## Abstract

Previous studies have shown that the congruency sequence effect (CSE) is usually domain-specific, that is, no transfer across different conflict tasks is observed. The goal of the present study was to test whether the lack of a CSE transfer across the Simon and Eriksen flanker tasks can be explained by a confound of conflict type and the temporal dynamics of conflict processing (i.e., the temporal overlap of target and distractor processing). By presenting the target in the Eriksen task before the distractors, we were able to largely align the temporal overlap of distractor-to-target processing (as indexed by delta plots) in the Simon and Eriksen tasks. Nevertheless, across four experiments we found little, if any, evidence for a transfer of the CSE across tasks. Overall, the results demonstrate that cognitive control is highly specific to the type of conflict, even when controlling for the temporal dynamics of conflict processing.

## Introduction

We constantly need to select and process task-relevant information in environments filled with distracting information. Adaptive goal-directed behavior requires sophisticated control mechanisms to shield target processing from potentially harmful distractions. To uncover these mechanisms in the laboratory, researchers often use various conflict tasks to study behavior. In these tasks, participants select a response based on a target dimension while ignoring a distractor dimension. For example, in the Simon task participants respond to a task-relevant feature (e.g., the color of a circle) while ignoring its location (e.g., Chen et al., 2021; Heuer & Wühr, 2024; Hommel, 2011; Simon & Rudell, 1967). In the Eriksen flanker task (e.g., Eriksen & Eriksen, 1974; Servant & Logan, 2019), participants respond to a centrally positioned task-relevant feature (e.g., the color of a circle) while ignoring the surrounding flankers (e.g., the colors of adjacent circles). Although these tasks involve different types of distractors (stimulus location or flanker identity), both produce robust congruency effects: Performance, measured by reaction time and error rates, is typically better when the response associated with the target and distractor matches (congruent trials) compared to when they do not match (incongruent trials).

### Domain-specific versus domain-general cognitive control in conflict tasks

One major question regarding cognitive control is the extent to which conflict resolution with different types of distractors relies on distinct (e.g., Egner, 2008; Zhu et al., 2025) or similar adaptation mechanisms (e.g., Freitas et al., 2007; Kan et al., 2013). A common approach to test for so-called domain-specific versus domain-general control mechanisms is to investigate the congruency sequence effect (CSE; e.g., Gratton et al., 1992; Luo & Proctor, 2022; Spinelli & Lupker, 2022). The CSE is typically seen as a marker of conflict adaptation and indicates that congruency effects are smaller after an incongruent trial compared to a congruent trial. For example, several researchers have argued that after experiencing conflict in incongruent trials, top-down control is upregulated by focusing more strongly on the target and/or stronger suppression of the distractor, leading to reduced congruency effects in the next trial (e.g., Botvinick et al., 2001). However, cognitive control accounts of the CSE have been challenged by memory-based explanations (e.g., Mayr et al., 2003), which attribute the CSE to the bottom-up formation of memory traces rather than top-down control. Specifically, in classic versions of conflict tasks with two response keys, stimuli and responses can repeat across trials, and the reactivation of any stimulus or response feature can automatically trigger the coactivation of associated feature codes. Since congruency and feature sequences are linked, with 50% of congruent-congruent and incongruent-incongruent sequences being direct repetitions (leading to faster responses), a CSE may emerge (at least in part) due to automatic (bottom-up) retrieval of specific features, rather than top-down control. To provide more conclusive evidence regarding cognitive control, confound-minimized paradigms are often used, which avoid stimulus and response repetitions across trials, thereby controlling for memory-related processes such as feature retrieval (e.g., Braem et al., 2019; Kelber et al., 2024a; Weissman et al., 2014).

Importantly, the scope (and its determinants) of conflict adaptation is still under debate. As reviewed by Braem et al. (2014), most theories of conflict adaptation assume or imply a task-specific (or conflict-type specific) scope of conflict adaptation, but it has also been suggested that the scope could be broader. For example, it has been suggested that conflict adaptation can strengthen all active representations (Verguts & Notebeart, 2008), raising the possibility of transfer across tasks when they are simultaneously active in working memory in the same experimental context (see also Braem et al., 2014; Zhu et al., 2025). Furthermore, several neuroimaging studies have identified similar domain-general (or multiple-demand) neural activation patterns when performing different tasks (e.g., Assem et al., 2020; Dosenbach et al., 2006; Peterson et al., 2002). Finally, consider also that there are a variety of control adjustments to improve conflict processing (e.g., Mittelstädt et al., 2023). For example, the experience of conflict may upregulate cognitive control by more strongly focusing on the target feature (i.e., target amplification) and/or by more strongly ignoring the distractor feature (i.e., distractor suppression). Thus, if, for example, target features are shared between two tasks, conflict resolution may be similarly affected with different distractors if control primarily amplifies target processing rather than suppressing the specific distractor.

Critically, while there are usually robust CSEs when the same conflict task repeats (e.g., Eriksen in trial n-1 and Eriksen in trial n), the CSE is usually absent when the conflict task switches (e.g., Eriksen in trial n-1 and Simon in trial n) (see, e.g., Bausenhart et al., 2021; Wendt et al., 2006; Wühr et al., 2015). Several studies using a range of different conflict tasks have replicated the finding that the CSE is specific to the type of conflict encountered in the previous trial (e.g., Egner, 2007; Funes et al., 2010; Lee & Cho, 2013), although a CSE may also occur even when some aspects of the task change (e.g., different response effectors, Weissman et al., 2015a, 2015b; different stimulus modalities, Lee & Cho, 2023). In fact, to our knowledge, there is only one study that has observed cross-task CSEs with both different distractors and targets using classic conflict tasks (i.e., between an Eriksen flanker and color- or spatial-Stroop task, Freitas et al., 2007). Some further evidence for domain-general control comes from a study that observed conflict adaptation across syntactic conflict (in a sentence reading task) and Stroop interference (Kan et al., 2013), and studies where both types of conflict are presented within a trial (e.g., interaction of Stroop and Simon effects in a hybrid conflict task, Weissman, 2020; but see Schlaghecken & Maylor, 2020, for an alternative interpretation of such interactions). However, we are not aware of any study that showed the transfer of the classic CSE across Simon and Eriksen tasks – that is, transfer across these two conflict tasks when people are faced with one of two possible and distinct sources of distracting information (i.e., stimulus location and flanker identity) in each trial.

The lack of a cross-task CSE between Simon and Eriksen tasks suggests that these tasks produce different types of conflict and hence require distinct conflict resolution mechanisms. In line with this suggestion, the location in the Simon task primarily produces motor-related conflict, and control processes might therefore suppress the location-triggered motor activation (e.g., Stürmer et al., 2002). While the flankers in the Eriksen task have also been shown to trigger irrelevant motor activation, they produce additional perceptual interference (e.g., Yeh & Eriksen, 1984), and control processes might therefore primarily operate by biasing spatial attention to the target (e.g., Scerif et al., 2006; Wendt et al., 2012), a control mechanism that is not helpful in dealing with conflict in the Simon task. Further support for domain-specific mechanisms comes from examining delta plots, which illustrate the size of congruency effects as a function of response speed. The slope of delta plots usually markedly differs across conflict tasks (i.e., rather decreasing slopes in the Simon task and rather increasing slopes in the Stroop and Eriksen flanker tasks, e.g., Ellinghaus et al., 2018; Kinoshita et al., 2017; Mittelstädt et al., 2022; Pratte et al., 2010), and it has been argued that these distinct delta plot patterns may arise because the mechanisms generating (and presumably resolving) conflict effects are different (e.g., Wiegand & Wascher, 2005).

### Temporal dynamics of target–distractor processing and their potential role in the congruency sequence effect

Critically, however, Ulrich et al., (2015, Diffusion Model for Conflict tasks) have demonstrated that the distinct delta plot patterns could simply result from differences in the relative temporal overlap of target-based and distractor-based activations. Specifically, the output of distractor processes follows a pulse-like function, meaning that the output of these processes first increases to a maximum and then decreases back to zero. This maximum is reached quickly for distractor activation produced by the irrelevant stimulus location in the Simon task, whereas it is reached relatively late for distractor activation produced by the irrelevant flankers in the Eriksen task. As a result, distractor activation already begins to decrease in the Simon task but continues to increase in the Eriksen task when superimposed with activation produced by target processing. In other words, different delta plot patterns may not arise due to different control mechanisms but rather due to differences in the temporal dynamics of distractor processing (i.e., faster processing of conflicting location than flanker information). Direct empirical support for this possibility comes from a study by Mackenzie et al., (2022; see also Hübner & Töbel, 2019; Mattler, 2003; Pratte, 2021). Specifically, they showed that when the onset of the target appeared delayed after flanker onset (i.e., flankers had a temporal head start), the delta plots of this delay-flanker Eriksen task were also decreasing, whereas when the target had a temporal head start (Mackenzie et al., 2022) or appeared with flanker onset (Hübner & Töbel, 2019; Mattler, 2003), the increasing delta plot characteristic of the Eriksen task was observed. Thus, these studies suggest that different delta plots in the Simon and Eriksen flanker tasks do not necessarily reflect domain-specific conflict processes and can instead be reconciled with a domain-general control mechanism.

First evidence for a potentially crucial role of the timing of distractor-to-target processes in the CSE comes from Weissman et al., (2014; see also Weissman et al., 2015a, 2015b). Specifically, a confound-minimized CSE was consistently observed in a prime-probe task when a distractor stimulus (e.g., a large arrow) was given a “head start” compared to the target stimulus (e.g., a small arrow) by presenting the distractor before the target (Weissman et al., 2014, 2015a, 2015b). However, while Weissman et al. (2014) observed a confound-minimized CSE in a Simon task (with vertical and horizontal locations), such a CSE was not observed in a color-word Stroop task or an Eriksen flanker task. Interestingly, though, when the distractor was presented before the target in the Eriksen flanker task, a CSE was observed – at least in conditions where the distractor letter was centrally presented before being replaced by the target. Therefore, Weissman et al. (2014) concluded that observing a confound-minimized CSE depends on faster stimulus-response (S-R) translation for the distractor than for the target, and suggested that future studies could investigate whether this is critical only in the previous trial, the current trial, or both.

While these previous studies have shown that timing is crucial when investigating the CSE within one particular conflict task, no study so far has explicitly considered the relative timing similarity as a boundary condition of the CSE across tasks with distinct types of distractors. Thus, previous reports of a lack of CSE transfer across tasks cannot be taken as strong evidence against domain-general control, but rather could reflect the fact that humans have difficulty making such adjustments across tasks with different temporal dynamics of conflict processing, regardless of whether identical or distinct types of distractors trigger conflict processing. The Simon and Eriksen flanker tasks appear to be ideal candidates for investigating this potential boundary condition, because, as mentioned above, both the Simon task and the Eriksen task with a flanker head start, seem to produce relatively faster distractor than target processing – a critical side condition to even observe a (confound-minimized) within-task CSE.^1^

### The present study

The goal of the present study was to provide a stronger test of domain-general conflict adaptation, examining whether a CSE can transfer across the Simon and Eriksen tasks when both tasks are matched in terms of relative timing of target and distractor processing, implying that domain-general control adjustments are tailored to the temporal dynamics of conflict processing. Since the distracting information is processed faster in the Simon (i.e., stimulus location) than in the Eriksen task (i.e., flanker identity), the corresponding conflict may primarily differ in timing rather than in the underlying control mechanism. With respect to the CSE, the control adjustment across trials may therefore be based (at least partially) on the timing of conflict processing in trial n-1. To investigate this possibility, we chose a design in which only one of the two conflict tasks occurs in each trial and the two tasks can alternate between trials. Others have used an integrated design in which both types of conflict occur within the same task (see Egner et al., 2007). Such an integrated design generally also allows CSEs to be examined within and across different conflict types. However, using such a design would not allow any conclusions regarding the question of whether conflict adaptation can transfer between different conflict tasks that are not integrated within the same task.

In four experiments, we experimentally aligned the temporal overlap of distractor and target processing between the Simon and Eriksen flanker tasks – two tasks with distinct types of distractors – and tested for transfer of the CSE across the two tasks. In Experiments 1 and 2, participants alternated between performing a standard Simon task and an Eriksen-like task on each trial (i.e., Simon, Eriksen, Simon, Eriksen…). In previous studies, this setup has typically not resulted in a CSE (e.g., Bausenhart et al., 2021). Critically, in half of the blocks, the Simon task alternated with an Eriksen task where the irrelevant flanker appeared before the relevant target (IR-Eriksen/Simon blocks). In the other half of the blocks, the Simon task alternated with an Eriksen task where the flanker appeared after the target (Experiment 1; RI-Eriksen/Simon blocks) or with the simultaneous onset of targets and distractors (Experiment 2; standard Eriksen/Simon blocks). Following previous studies (e.g., Mackenzie et al., 2022), we expected decreasing delta plots in the Simon task, and increasing delta plots in the Eriksen task when targets have a head start (i.e., RI-Eriksen in Experiment 1) or appear simultaneously with targets (i.e., standard Eriksen in Experiment 2). However, the delta plots should show a less pronounced increase (or possibly even a decrease) in the Eriksen task when flankers have a head start (i.e., IR-Eriksen; see Mackenzie et al., 2022), thereby making the timing between processing of target and distractor information more similar to the Simon task. While Experiments 1 and 2 investigated CSEs using two-alternative forced-choice (2-AFC) tasks (which may reflect lower memory-based and/or higher control-based adaptations; see Hommel et al., 2004; Mayr et al., 2003), in Experiments 3 and 4 we investigated CSEs controlled for stimulus repetitions (i.e., confound-minimized CSEs, which only reflect higher control-based adaptations; see Braem et al., 2019; Schmidt & Weissman, 2014) across tasks using four-alternative forced-choice (4-AFC) tasks exclusively in IR-Eriksen/Simon blocks. In Experiment 3, the two tasks again alternated on each trial, whereas in Experiment 4 the tasks were randomly intermixed, allowing us to also investigate within-task CSEs for both the Simon and Eriksen tasks.

Under the presumption that domain-general control adjustments are tailored to the temporal dynamics of conflict processing, the CSE (i.e., a smaller congruency effect after incongruent than congruent trials) should transfer between the IR-Eriksen and Simon tasks, as the temporal dynamics of conflict processing should be similar for these two tasks (as indexed by similar delta plots). A transfer of the CSE between the two tasks, however, should not occur (or to a lesser degree) for the RI-Eriksen and the standard Eriksen task, as in this case the temporal dynamics of conflict processing should considerably differ between the two tasks (as indexed by different delta plots). In contrast, if the CSE is due to domain-specific control processes, there should be no transfer of the CSE, irrespective of the timing manipulation in the Eriksen task. Thus, we would expect only within-task CSEs (Simon and IR-Eriksen) and no transfer between the Simon and Eriksen tasks.

## Experiment 1

In Experiment 1, we investigated the CSE between a standard Simon task and an Eriksen task where the irrelevant flanker appeared either before (IR-Eriksen) or after (RI-Eriksen) the relevant target. The two task types were presented in separate blocks, IR-Eriksen and RI-Eriksen blocks. Within each block, tasks alternated trial-wise (e.g., in RI-Eriksen blocks: trial 1 = RI-Eriksen; trial 2 = Simon; trial 3 = RI-Eriksen; trial 4 = Simon and so on). To further increase predictability, participants were explicitly instructed about the sequence in each block. For both tasks, participants were instructed to respond to the target (i.e., identifying whether the letter was H or S) while ignoring the distractor (i.e., the location in the Simon task and the flankers in the Eriksen task). Under the additional assumption that control adjustments are tailored to the temporal dynamics of conflict processing, the domain-general control hypothesis predicts a three-way interaction between block type (IR-Eriksen vs. RI-Eriksen), previous congruency, and current congruency, indicating a transfer of the CSE (interaction between previous and current congruency) in IR-Eriksen blocks but not in RI-Eriksen blocks, provided that the delta plots for the Simon and Eriksen task can be aligned in IR-Eriksen blocks by giving the flankers a head start.

### Method

#### Participants

One hundred participants were tested but data of 17 participants were excluded because of excessively high error rates (> 25% error trials).^2^

#### Apparatus and stimuli

The experiment was conducted online using the JavaScript library jsPsych (de Leeuw et al., 2023). All stimuli were presented in black on a gray background. A centrally positioned black plus sign (+) served as the fixation point. For each participant, the stimulus letters (H vs. S) were randomly assigned to left- and right-hand responses. In Simon task trials, the target letter appeared approximately to the left or right of the center of the screen (measured to the center of the letter). In the Eriksen task blocks, the target letter was centrally presented and two flanker letters appeared on each side of the target letter (e.g., HHSHH). Responses were key presses with the left and right index fingers on the “Q” and “P” keys of a QWERTZ computer keyboard.

#### Procedure

Overall, participants were tested in 12 experimental blocks consisting of 80 trials. Half of the participants were tested with six RI-Eriksen blocks followed by six IR-Eriksen blocks, whereas this order was reversed for the other half of participants. The specific task for the first trial in each block was randomly selected. In advance of each block, participants were informed about (a) the upcoming task requirements (e.g., RI-Eriksen: Respond to the identity of the first presented central target letter and ignore the following flanker letter vs. Simon: Respond to the lateralized target letter and ignore its location) and (b) they were also told that the two task types alternated trial-wise and they can/should use the predictability to prepare. At the beginning of each trial, a fixation cross was presented for 500 ms. For a Simon task trial, a single letter was then presented to the left or right of the screen. For an RI-Eriksen task trial, a target was presented on the screen for 150 ms, followed by the onset of two flanking letters on each side. For an IR-Eriksen task trial, the flankers were first presented for 150 ms, followed by the onset of the targets. After each correct trial, a blank screen was presented for 500 ms before the next trial started whereas after incorrect (or too slow trials) an additional error screen was presented for 1 s if the response was incorrect or too slow (> 2 s).

### Results

Data were analyzed using R, Version 4.3.2 by R Core Team (2023), and the R package DMCfun (Mackenzie & Dudschig, 2021). The R package ggplot2 (Wickham, 2016) was used to create figures (with the exception of delta plots). The first block of each block type was excluded from all analyses. For both percentage error (PE) and RT analyses, we excluded “too-slow” (0.2%) and “too-fast” (< 150 ms, 0.4%) trials, as well as post error trials (5.1%). For RT analyses, we additionally excluded choice error trials (4.6 % of remaining trials).

#### Delta plots

**Fig. 1:**
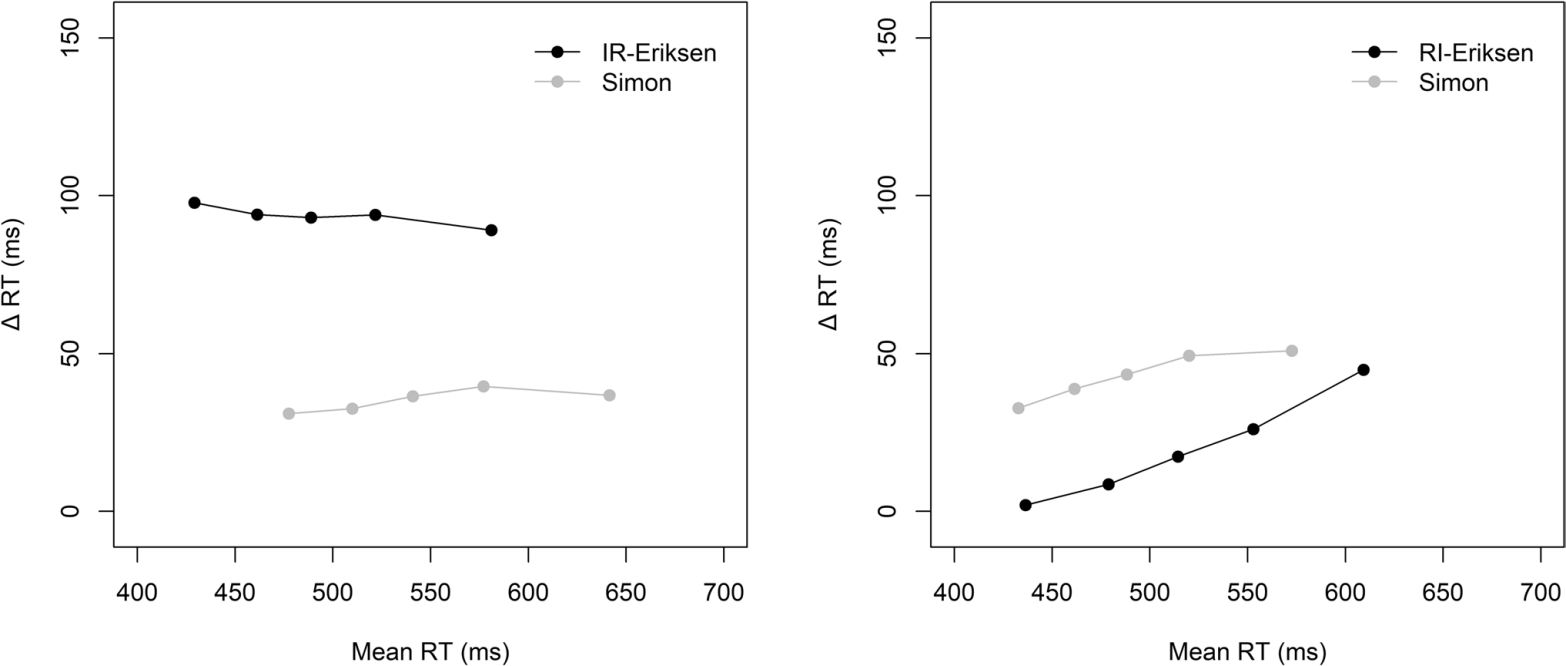
Delta plots showing the incongruent minus congruent differences in mean reaction time (Δ RT) within each of five RT percentiles, plotted against the quantile average RTs separately for each block type (IR-Eriksen vs. RI-Eriksen) as a function of task in Experiment 1

Figure 1 shows the delta plots for the two block types. An ANOVA with the within-subject factors block type (IR-Eriksen vs. RI-Eriksen) and current task (Simon vs. Eriksen) revealed a significant main effect of block type reflecting overall less positive slopes in IR-Eriksen (0.01) than RI-Eriksen blocks (0.17), *F*(1, 82) = 41.64, *p* <.001, η_p_^2^ =.34. The main effect of task was not significant (*F* < 1), but the interaction was, *F*(1, 82) = 10.29, *p* =.002, η_p_^2^ =.11. The delta plot of the Eriksen task was less increasing (and even negative) in IR-Eriksen than in RI-Eriksen blocks (−0.05 vs. 0.20; *p* <.001). Interestingly, the Simon task delta plot was also less increasing in IR-Eriksen than in RI-Eriksen blocks (0.06 vs. 0.14; *p* =.018), albeit the difference was less pronounced than for the Eriksen task. When comparing the two delta plots within block types, their slopes differed significantly in IR-Eriksen blocks (*p* =.003), but not in RI-Eriksen blocks (*p* =.103).

### Mean reaction time (RT) and percentage error (PE)

**Fig. 2:**
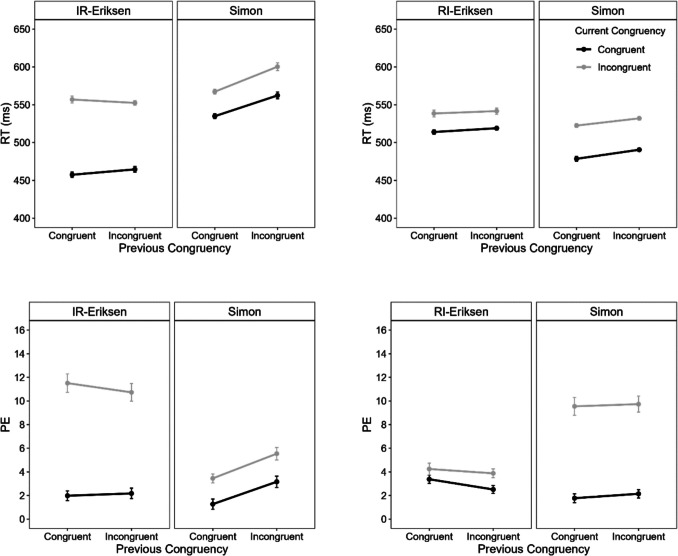
Mean reaction time (RT) and percentage errors (PE) as a function of current congruency (congruent vs. incongruent), previous congruency and task (Eriksen vs. Simon) separately for IR-Eriksen (flankers presented before target; left) and RI-Eriksen (target presented before flankers; right) blocks in Experiment 1. Error bars represent ± 1 within-subject *SE* (Morey, 2008)

An overall ANOVA with the within-subject factors current congruency, previous congruency, current task (Eriksen vs. Simon), and block type (IR-Eriksen vs. RI-Eriksen) revealed a significant four-way interaction for mean RT, *F*(1, 82) = 5.23, *p* =.025, η_p_^2^ =.06, but not for PE, *F*(1, 82) = 1.78, *p* =.186, η_p_^2^ =.02. Importantly, the three-way interaction between block type, previous congruency, and current congruency was not significant, for either mean RT, *F*(1, 82) = 0.04, *p* =.850, η_p_^2^ <.01, or PE, *F*(1, 82) = 0.70, *p* =.406, η_p_^2^ =.01, and there was no evidence for a CSE overall (interaction between current and previous congruency), for either mean RT, *F*(1, 82) = 1.92, *p* =.170, η_p_^2^ =.02, or PE, *F*(1, 82) = 0.13, *p* =.720, η_p_^2^ <.01. To further explore the four-way interaction, in the next step we analyzed RT and PE performance separately for each block type. Figure 2 shows mean RT and PE performance separately for the two block types.

#### Mean RT and PE in IR-Eriksen blocks

ANOVAs with the within-subject factors current congruency, previous congruency and current task (IR-Eriksen vs. Simon) were conducted on mean RT and PE. Note that when the current task was IR-Eriksen (Simon), the previous task was Simon (IR-Eriksen) as task changed every trial. These ANOVAs revealed a significant three-way interaction for RT, *F*(1, 82) = 9.35, *p* =.003, η_p_^2^ =.10, but not for PE, *F*(1, 82) = 2.21, *p* =.141, η_p_^2^ =.03. To further examine the three-way interaction, we conducted ANOVAs with the factors current and previous congruency separately for each task.

When the current task was the IR-Eriksen task (and the previous Simon), there was a significant main effect of current congruency, *F*(1, 82) = 475.19, *p* <.001, η_p_^2^ =.85, and a significant interaction for mean RTs, *F*(1, 82) = 7.22, *p* =.009, η_p_^2^ =.08. The IR-Eriksen flanker effect was smaller after incongruent (88 ms) than after congruent Simon trials (99 ms), reflecting a CSE of 12 ms. The main effect of previous congruency was not significant, *F* < 1. For PEs, the main effect of congruency was significant, *F*(1, 82) = 91.47, *p* <.001, η_p_^2^ =.53 The IR -Eriksen flanker effect was descriptively slightly smaller after incongruent (8.6%) than after congruent Simon trials (9.5%) reflecting a CSE of 1.0%. The main effect of previous congruency, *F* < 1, and the interaction, *F*(1, 82) = 2.12, *p* =.149, η_p_^2^ =.03, were not significant.

When the current task was the Simon task (and the previous IR-Eriksen), there were significant main effects of current congruency, *F*(1, 82) = 144.69, *p* <.001, η_p_^2^ =.64, and previous congruency, *F*(1, 82) = 43.14, *p* <.001, η_p_^2^ =.34, but no significant interaction for mean RTs, *F*(1, 82) = 2.19, *p* =.143, η_p_^2^ =.03. The Simon effect was descriptively even larger after incongruent (38 ms) than after congruent IR-Eriksen trials (32 ms) reflecting a reversed CSE of −6 ms. For PE, there were also significant main effects of current congruency, *F*(1, 82) = 30.57, *p* <.001, η_p_^2^ =.27, and previous congruency, *F*(1, 82) = 13.53, *p* <.001, η_p_^2^ =.14, but also no significant interaction, *F*(1, 82) = 0.12, *p* =.725, η_p_^2^ <.01.

#### Mean RT and PE in RI-Eriksen blocks

The three-way interaction was not significant for RT, *F*(1, 82) = 0.01, *p* =.917, η_p_^2^ <.01, or for PE,* F*(1, 82) = 0.44, *p* =.510, η_p_^2^ =.01. For the sake of comparison, we nevertheless conducted and report the results of the ANOVAs separately for each task.

When the current task was the Simon task (and the previous RI-Eriksen), there were significant main effects of current congruency, *F*(1, 82) = 390.93, *p* <.001, η_p_^2^ =.83, and previous congruency,* F*(1, 82) = 18.21, *p* <.001, η_p_^2^ =.18, but no interaction for mean RT,* F*(1, 82) = 0.50, *p* =.481, η_p_^2^ =.01. For PE, the main effect of current congruency was significant, *F*(1, 82) = 82.08, *p* <.001, η_p_^2^ =.50. The main effect of previous congruency, *F*(1, 82) = 0.53, *p* =.468, η_p_^2^ =.01, and the interaction,* F*(1, 82) = 0.07, *p* =.797, η_p_^2^ <.01, were not significant.

When the current task was the RI-Eriksen task (and the previous Simon), there were significant main effects of current congruency, *F*(1, 82) = 21.06, *p* <.001, η_p_^2^ =.20, and previous congruency,* F*(1, 82) = 4.02, *p* =.048, η_p_^2^ =.05, but no significant interaction for mean RT, *F*(1, 82) = 0.24, *p* =.627, η_p_^2^ <.01. For PE, the main effect of current congruency was significant, *F*(1, 82) = 6.50, *p* =.013, η_p_^2^ =.07. The main effect of previous congruency, *F*(1, 82) = 3.40, *p* =.069, η_p_^2^ =.04, and the interaction, *F*(1, 82) = 0.60, *p* =.442, η_p_^2^ =.01, were again not significant.

### Discussion

We were successful in altering the delta plot for the Eriksen flanker task by manipulating the timing of target and distractor onsets, as the slopes were less increasing in the IR than in the RI-Eriksen condition and appeared to rather follow a similar time-course as in the Simon task. It should, however, be noted that in IR-Eriksen blocks the slopes between the delta plots of the two tasks still differed significantly, although these differences were only numerically small (as can also be seen in Fig. 2, where the slopes appear visually quite similar). In this context, it is particularly surprising that the Simon task delta plot was not decreasing, but rather remained stable in IR-Eriksen blocks or even increased in RI-Eriksen blocks. The evidence concerning domain-general control was mixed. Although the overall analysis indicated no CSE and no modulation of the CSE due to block type, the significant four-way interaction suggested a unidirectional CSE only in IR-Eriksen blocks from the Simon to the IR-Eriksen task and not vice versa.

## Experiment 2

Experiment 1 provided inconclusive evidence for domain-general control, as in this case one would have expected a bidirectional rather than a unidirectional CSE between the Simon and the IR-Eriksen task. Since this pattern of results still provides (at least partial) evidence for a cross-task CSE, we conducted another experiment to determine whether it can be replicated. Thus, in Experiment 2 we again included blocks where the IR-Eriksen task alternated with the standard Simon task (IR-Eriksen blocks). To further ensure that a potential transfer is specific to the aligned temporal dynamics of conflict processing in the Simon and IR-Eriksen tasks and could not also emerge with the standard Eriksen task (without temporal offset between target and flankers), we also included blocks with a Simon task alternating with a standard Eriksen flanker task (Eriksen blocks) instead of the RI-Eriksen task as in Experiment 1.

### Method

#### Participants

As in Experiment 1, 100 participants were tested but data of four participants were excluded because of excessive error rates (> 25% error trials).

#### Apparatus, stimuli, and procedure

The setup of Experiment 2 was identical to Experiment 1, except that the RI-Eriksen condition was replaced with a standard Eriksen task, in which the flanker stimuli appeared simultaneously with the target.

### Results

As in Experiment 1, and following our preregistration, the first block of each block type was excluded from all analyses, as were “too-slow” (0.3%) and “too-fast” (< 150 ms, 0.3 %) trials, as well as post error trials (5.3%) and the first trial of each block. For RT analyses, we additionally excluded choice error trials (4.7% of remaining trials).

#### Delta plots

**Fig. 3:**
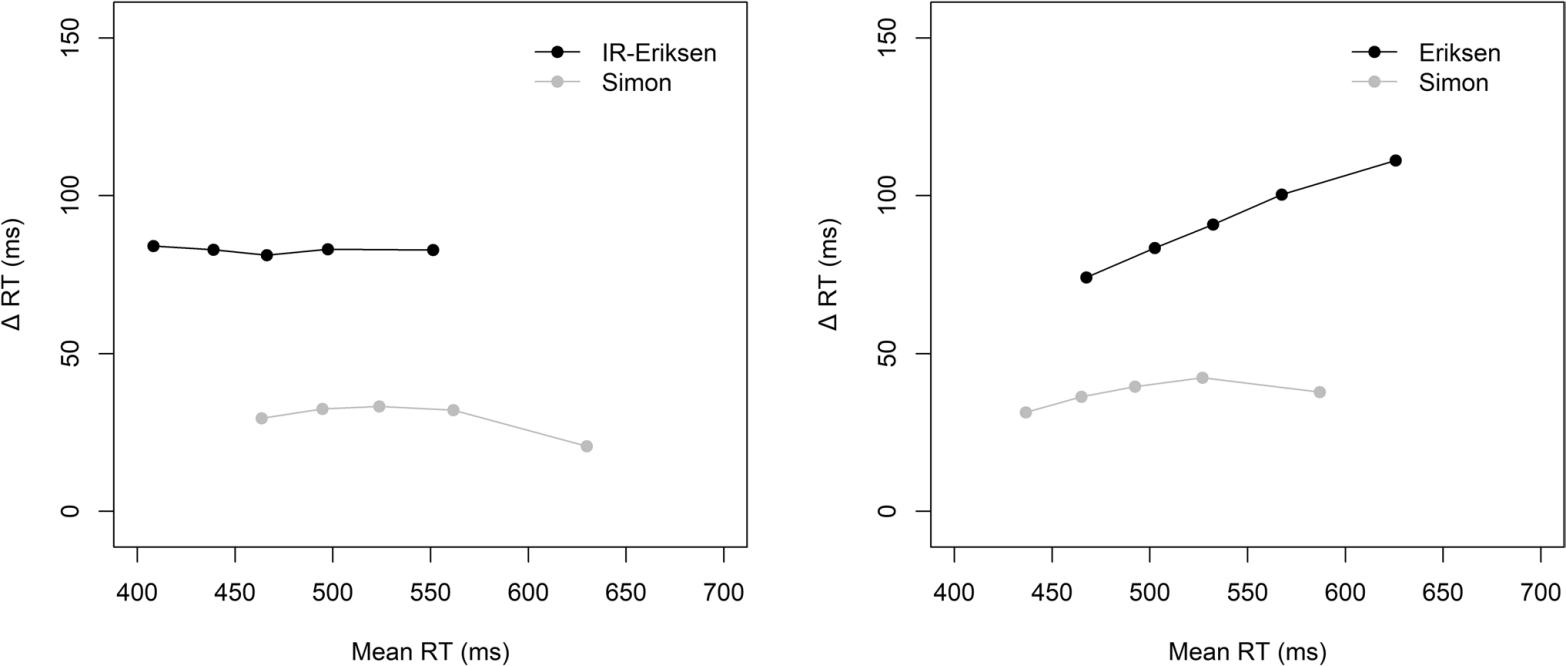
Delta plots showing the incongruent minus congruent differences in mean reaction time (Δ RT) within each of five RT percentiles, plotted against the quantile average RTs separately for each block type (IR-Eriksen vs. Eriksen) as a function of task in Experiment 2

Figure 3 shows the delta plots for the two block types. There were again similar (stable) delta plots for the Simon and IR-Eriksen conditions in the IR-Eriksen blocks. However, in the standard Eriksen blocks, the Eriksen delta plot was increasing, whereas the Simon delta plot was again rather stable. An ANOVA with the within-subject factors block type (IR-Eriksen vs. Eriksen) and current task revealed a significant main effect block type reflecting overall less positive slopes in IR-Eriksen (−0.02) than Eriksen blocks (0.15), *F*(1, 95) = 56.38, *p* <.001, η_p_^2^ =.37. The main effect of task was also significant, *F*(1, 95) = 28.95, *p* <.001, η_p_^2^ =.23, reflecting less positive slopes in the Simon (< −0.01) than the Eriksen flanker task (0.13), *F*(1, 95) = 28.95, *p* <.001, η_p_^2^ =.23. The interaction was also significant, *F*(1, 82) = 13.27, *p* =.001, η_p_^2^ =.12. The delta plot of the flanker task was less increasing in IR-Eriksen than Eriksen blocks (< −0.01 vs. 0.26; *p* <.001). Interestingly, the Simon task was also less increasing (and negative) in IR-Eriksen than Eriksen blocks (−0.04 vs. 0.04; *p* =.016), albeit the difference was less pronounced than for the Eriksen task. Comparing the Eriksen and Simon tasks within the two block conditions indicated that the Eriksen delta plot differed significantly from the Simon delta plot in Eriksen blocks (0.26 vs. 0.04, *p* <.001), whereas they did not differ in IR-Eriksen blocks (< −0.01 vs. −0.04, *p* =.144).

#### Mean RT and PE

**Fig. 4:**
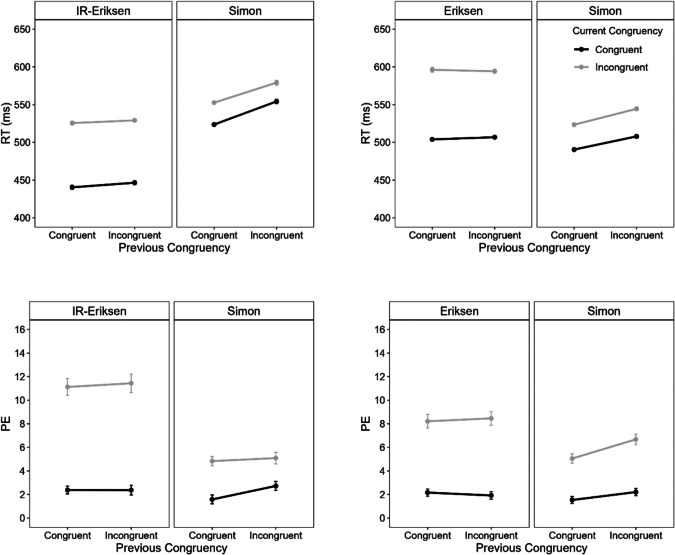
Mean reaction time (RT) and percentage errors (PE) as a function of current congruency (congruent vs. incongruent), previous congruency and current task (Eriksen vs. Simon) separately for IR-Eriksen (left) and standard Eriksen (right) blocks in Experiment 2. Error bars represent ± 1 within-subject *SE* (Morey, 2008)

Overall ANOVAs with the within-subject factors current congruency, previous congruency, current task (Eriksen vs. Simon), and block type (IR-Eriksen vs. Eriksen) revealed no significant four-way interaction for mean RT, *F*(1, 95) = 1.55, *p* =.216, η_p_^2^ =.01, or PE, *F*(1, 95) = 1.33, *p* =.251, η_p_^2^ =.01. There was also no significant three-way interaction between block type, current congruency and previous congruency, neither for mean RT, *F*(1, 95) = 0.48, *p* =.489, η_p_^2^ =.01, nor for PE, *F*(1, 95) = 2.41, *p* =.124, η_p_^2^ =.02, and there was again also no evidence for a CSE, either for mean RT, *F*(1, 95) = 1.01, *p* =.317, η_p_^2^ =.01, or for PE, *F*(1, 95) = 0.38, *p* =.537, η_p_^2^ <.01. For mean RT, all other main effects and two-way interactions were significant, *p*s <.005 (all other three-way interactions, *p*s >.172). For PE, all main effects were significant, *p*s <.015. Additionally, there were significant interactions between current congruency and current task, *F*(1, 95) = 48.09, *p* <.001, η_p_^2^ =.34, previous congruency and current task, *F*(1, 95) = 5.75, *p* =.018, η_p_^2^ =.06, current task and block type, *F*(1, 95) = 17.31, *p* <.001, η_p_^2^ =.15, and a significant three-way interaction between congruency, current task and block type, *F*(1, 95) = 13.07, *p* <.001, η_p_^2^ =.12, all other *p*s >.185. Figure 4 shows mean RT and PE performance separately for the two block types.^3^

### Discussion

As in Experiment 1, we were able to alter the slope of the delta plot for the Eriksen flanker task, with the slope increasing less in the IR-Eriksen than in the standard Eriksen condition. Importantly, although the delta plot of the IR-Eriksen task largely matched the delta plot of the Simon task, there was no evidence for a cross-task CSE, neither from the IR-Eriksen to the Simon task nor from the Simon to the IR-Eriksen task. Thus, Experiment 2 provided no support for domain-general control. Instead, the domain-specificity of the CSE appears to persist even when the timing of the interaction between distractor and target processing is aligned between the two tasks. Since Experiment 2 did not replicate the partial cross-task CSE observed in Experiment 1, we decided to conduct another experiment with the aim of further optimize the conditions for a potential domain-general CSE.

## Experiment 3

In Experiments 1 and 2, we employed the classic 2-AFC versions of the Eriksen flanker and Simon tasks. However, with this paradigm, any potentially observed CSE between trials could have been attributed primarily to lower-level memory processes, such as stimulus and/or response repetitions, rather than higher-level cognitive control mechanisms. To more directly test trial-by-trial cognitive control adjustments, it is beneficial to use a design that minimizes memory-based processes and encourages control adaptation. Possibly, participants only engage in cognitive control adaptation if they cannot rely on lower-level memory learning processes (see Bugg, 2014). For example, Spinelli and Lupker (2023) highlighted that a proportion congruency effect in contingency-free transfer items was usually not observed, unless contingency learning was also ruled out for the inducer items. Furthermore, using an auditory prime-probe conflict task, Kelber et al. (2024b) demonstrated that the CSE primarily transfers between different speakers when a confound-minimized design is used, but not in a confounded design (as in the current Experiments 1 and 2). Thus, domain-general control might only be implemented in situations where higher-level control is clearly required. Therefore, in Experiment 3 we used a 4-AFC, confound-minimized design that eliminated stimulus and response repetitions between trials. As in the previous experiments, the Simon task alternated with the IR Eriksen task, but each task had distinct stimuli (e.g., Simon: E and O; Eriksen: W and P) and was mapped to different response effectors (e.g., Simon: index fingers, Eriksen: middle fingers).

### Method

#### Participants

As in Experiments 1 and 2, and following our preregistration, 100 participants were tested but data of two participants were excluded due to > 25% error trials.

#### Apparatus, stimuli and procedure

The setup of Experiment 3 was identical to that of Experiments 1 and 2, with the exception that we used blocks in which a Simon task alternated with an IR-Eriksen task within a confound-minimized 4AFC paradigm. The stimulus letters were W, E, O, and P. Participants responded with key presses using their left and right index and middle fingers on the “W,” “E,” “O,” and “P” keys of a QWERTZ keyboard. To prevent repetition of distractors, targets, and correct responses between consecutive trials, we created two distinct stimulus-response sets: the index-finger set included the letters “E” and “O,” while the middle-finger set consisted of “W” and “P.” For each participant, it was randomly determined whether the index- or middle-finger set would be used for the Simon or Eriksen task. As in the previous experiments, trials alternated between the Simon and Eriksen tasks in each block, ensuring no repetitions due to the unique stimuli for each task. The stimulus-onset asynchrony (SOA) for the flanker head start was again set at 150 ms, and the response deadline was extended from 2,000 ms to 2,500 ms to account for the increased difficulty of the task. Participants completed nine experimental blocks, each consisting of 80 trials. The specific task for the first trial in each block was randomly selected, and the instructions were consistent with those from the previous experiments. Additionally, after each correct trial, a blank screen was presented for 500 ms before the next trial began, while an error screen was displayed for 1 s following incorrect or excessively slow responses.

### Results

Following our preregistration, the first two blocks were excluded from any analyses. For both mean RT and PE analyses, we excluded “too-slow” (1.2%) and “too-fast” (< 150 ms, 0.8%) trials as well as post-error trials (6.3%) and the first trial of each block. For RT analyses, we additionally excluded choice error trials (4.5% of remaining trials).

#### Delta plots

**Fig. 5:**
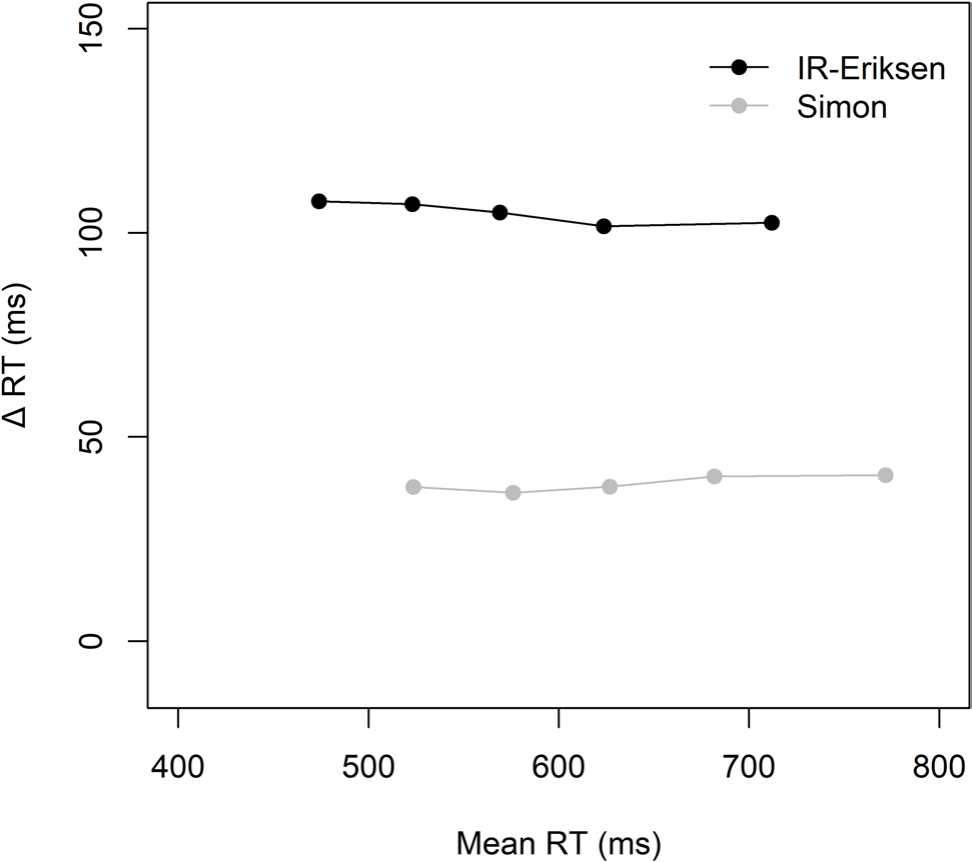
Delta plot showing the incongruent minus congruent differences in mean reaction time (Δ RT) within each of five RT percentiles, plotted against the quantile average RTs as a function of task in Experiment 3

The slopes for the two tasks were quite similar, showing a rather stable pattern across the RT distributions (Simon: 0.02, IR-Eriksen: −0.04; see Fig. 5). Nevertheless, the slight difference between the two slopes almost reached significance, *t* (97) = 1.92, *p* =.058, *d* = 0.19.

#### Mean RT

**Fig. 6:**
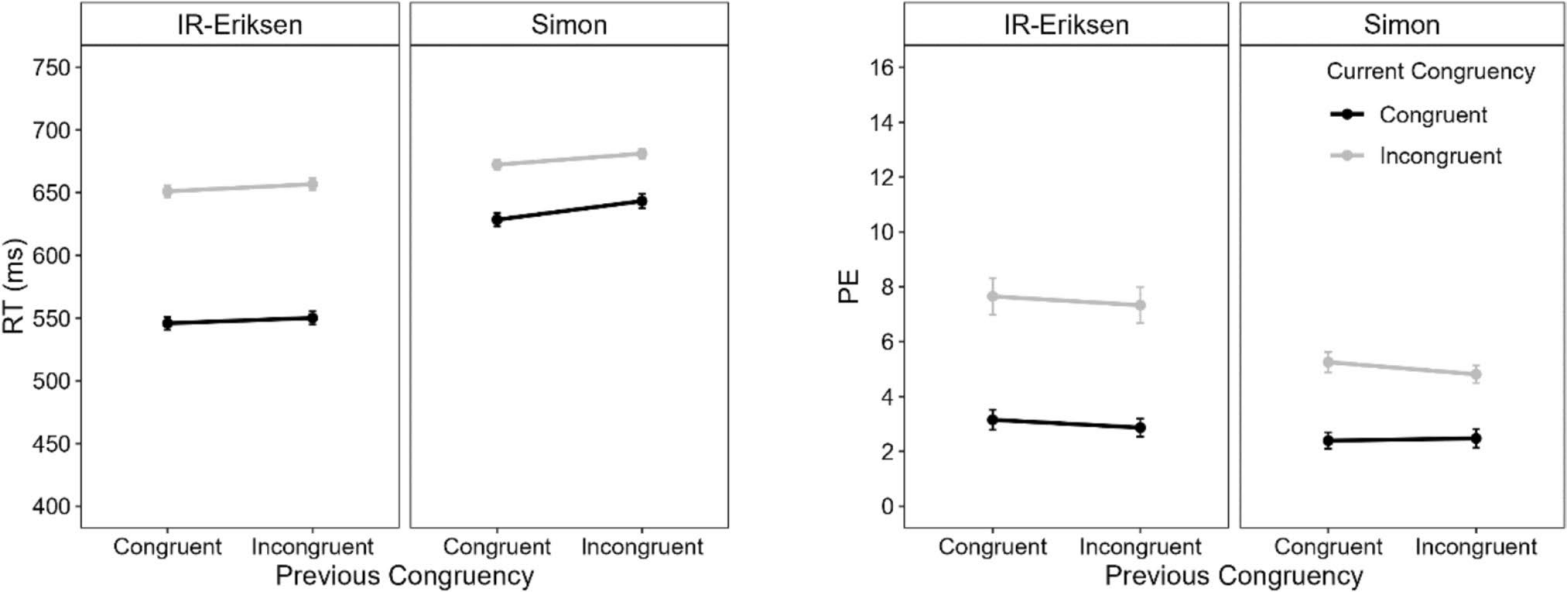
Mean reaction time (RT) and percentage errors (PE) as a function of current congruency (congruent vs. incongruent), previous congruency, and task (IR-Eriksen vs. Simon) in Experiment 3. Error bars represent ± 1 within-subject *SE* (Morey, 2008)

Figure 6 (left panel) shows mean RT as a function of current congruency, previous congruency, and task. An ANOVA with the within-subject factors current congruency, previous congruency and current task showed that all main effects were significant. The main effect of current congruency indicated faster responses in congruent than incongruent trials (592 vs. 665 ms), *F*(1, 97) = 719.50, *p* <.001, η_p_^2^ =.88. Responses were also faster after congruent than after incongruent trials (624 vs. 634 ms), *F*(1, 97) = 21.81, *p* <.001, η_p_^2^ =.18. The main effect of task indicated faster responses in the IR-Eriksen than in the Simon task (601 vs. 656 ms), *F*(1, 97) = 57.43, *p* <.001, η_p_^2^ =.37. A significant interaction between current congruency and task reflected a larger congruency effect in the IR-Eriksen than in the Simon task (106 vs. 26 ms), *F*(1, 97) = 92.18, *p* <.001, η_p_^2^ =.49. A significant interaction between previous congruency and current task indicated that RTs differed more between previous congruent and incongruent trials when the current task was the Simon (650 vs. 662 ms) than when it was the IR-Eriksen task (598 vs. 603 ms), *F*(1, 97) = 4.71, *p* =.032, η_p_^2^ =.05. Critically, neither the two-way interaction between current and previous congruency, *F* < 1, nor the three-way interaction was significant, *F*(1, 97) = 1.24, *p* =.268, η_p_^2^ =.01. To ease the comparison with the results of the previous experiments, we additionally conducted ANOVAs with the factors current and previous congruency, separately for each task. Again, there was no significant CSE for either the Simon, *F*(1, 97) = 1.40, *p* =.240, η_p_^2^ =.01, or the IR-Eriksen task, *F* < 1.

#### PE

Figure 6 (right panel) shows PE as a function of current congruency, previous congruency, and task. The ANOVA on PE revealed a significant main effect of current congruency, *F*(1, 97) = 54.30, *p* <.001, η_p_^2^ =.36, with larger error rates in incongruent than congruent trials (6.3 vs. 2.7%). The significant main effect of current task reflected more errors in the IR-Eriksen than in the Simon task (5.2 vs. 3.7%), *F*(1, 97) = 13.61, *p* <.001, η_p_^2^ =.12. The main effect of previous congruency was not significant, *F*(1, 97) = 2.30, *p* =.133, η_p_^2^ =.02. Finally, there was a significant interaction between current congruency and task, *F*(1, 97) = 4.95, *p* =.028, η_p_^2^ =.05, with a larger congruency effect in the Eriksen than in the Simon task (4.5 % vs. 2.6 %). All other interactions were not significant, *F*s < 1. Separate ANOVAs for each task also showed no significant CSE for either the Simon, *F*(1, 97) = 1.20, *p* =.276, η_p_^2^ =.01, or the IR-Eriksen task, *F* < 1.

### Discussion

The results of Experiment 3 again provide no evidence for domain-general control even under optimized conditions (i.e., by excluding both differences in the temporal dynamics of conflict processing and stimulus repetitions as potentially confounding factors), as there was no CSE when alternating between a Simon task and an Eriksen flanker task with a flanker head start in a confound-minimized paradigm. Instead, the results support a domain-specific control account of the CSE, even when the timing of distractor processing is matched between tasks (i.e., there was no significant difference in delta plot slopes between the Simon and IR-Eriksen tasks) and low-level confounds are controlled for. However, in such a confound-minimized design, even when only using a single conflict task (and thus investigating the CSE within the same task), the CSE may only be present (or particularly evident) when certain side conditions are met (see Weissman et al., 2014). Consequently, we decided to conduct one final experiment with a modified confound-minimized design.

## Experiment 4

In a previous study investigating the CSE within a confound-minimized only-flanker task, a CSE was observed only when a distractor letter preceding the target array was also presented at a central location (Weissman et al., 2014). Although a CSE in a confound-minimized flanker task has also been reported without a central distractor (using only flankers as in Experiment 3), including a central distractor may be beneficial, as it increases overall congruency effects and thus probably also the potential for conflict adaptation (see Bognar et al., 2024; Botvinick et al., 2001). Additionally, using a flanker array with a central distractor (i.e., HHHHH instead of HH_HH) necessitates implementing a blank screen before the presentation of targets and distractors. Such blank screens are typically used in confound-minimized prime-probe tasks to allow control processes to inhibit distractor-based activation before target processing begins (e.g., Weissman et al., 2014). Therefore, these modifications (the addition of a central distractor and a blank screen) were also implemented in Experiment 4. Furthermore, to demonstrate that our paradigm can produce confound-minimized CSEs across the same task, the two conflict tasks were presented in random order so that sequences in which the same conflict task repeated could also be analyzed. Note, however, that while there were no stimulus and response repetitions for the Simon task, the location was always horizontal (i.e., and hence could repeat). A purely confound-free CSE between Simon task trials would have required the additional implementation of a vertical location to avoid location repetitions, thereby complicating the task.

### Method

#### Participants

Following our preregistration, 100 participants were tested, but the data of four participants were excluded because of excessively high error rates (> 25% error trials).

#### Apparatus, stimuli, and procedure

The setup of Experiment 4 was identical to that of Experiments 3 except for the following changes. First, in the IR-Eriksen task, the distractor array included a central distractor (e.g., WWWWW), which was presented for 100 ms, followed by a 50-ms blank screen before both the target and the distractors appeared. Second, we employed two stimulus- and effector-specific Simon tasks (e.g., Simon-index: E and O with index fingers; Simon-middle: W and P with middle fingers) and two stimulus- and effector-specific IR-Eriksen tasks using the corresponding stimuli and response effectors from the Simon task (e.g., Eriksen-index: E and O with index fingers; Eriksen-middle: W and P with middle fingers). As in Experiment 3, specific stimulus-response pairs alternated across trials (i.e., trial-1: index-finger response, trial-2: middle-finger response, trial-3: index-finger response, trial-4: middle-finger response…), but it was randomly determined whether participants would perform the IR-Eriksen task or the Simon task. Thus, this design allowed us to measure a confound-minimized CSE both within (i.e., between Eriksen-index and Eriksen-middle and between Simon-index and Simon-middle) and across tasks (i.e., CSE between Simon-index and Eriksen-middle or Eriksen-index and Simon-middle). Except for the addition of one extra block (making a total of nine blocks) and an extension of the response deadline (3,000 ms instead of 2,500 ms), all other aspects (e.g., trial numbers) were identical to Experiment 3.

### Results

We applied the same data preparation procedure as in Experiment 3. Accordingly, the first two blocks were excluded from any analyses. For both PE and RT analyses, we excluded “too-slow“ (1.9%) and “too-fast” (< 150 ms, 0.6%) trials as well as post-error trials (9.0%) and the first trial of each block. For RT analyses, we additionally excluded choice error trials (5.9% of remaining trials).

#### Delta plots

**Fig. 7:**
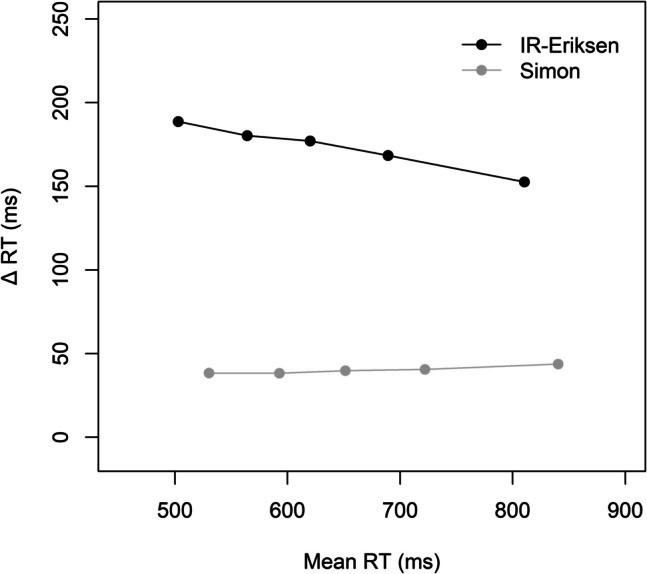
Delta plot showing the incongruent minus congruent differences in mean reaction time (Δ RT) within each of five RT percentiles, plotted against the quantile average RTs as a function of task in Experiment 4

As in Experiment 3, the delta plot of the IR-Eriksen task was more negative than the delta plot of the Simon task (−0.11 vs. 0.02; see Fig. 7), *t* (95) = 4.27, *p* <.001, *d* = 0.44.

#### Mean RT and PE

Separate ANOVAs with the within-subject factors current congruency, previous congruency, current task (IR-Eriksen vs. Simon) and previous task (IR-Eriksen vs. Simon) were first conducted on mean RT and PE. For mean RT, the four-way interaction was significant, *F*(1, 95) = 7.04, *p* =.009, η_p_^2^ =.07, whereas it did not reach significance for PE, *F*(1, 95) = 3.64, *p* =.059, η_p_^2^ =.04. The three-way interaction between current task, current congruency and previous congruency was not significant, either for mean RT, *F*(1, 95) = 0.08, *p* =.782, η_p_^2^ <.01, or for PE, *F*(1, 95) = 1.09, *p* =.299, η_p_^2^ =.01. However, the interaction between current and previous congruency was significant for mean RT, *F*(1, 95) = 20.53, *p* <.001, η_p_^2^ =.11, but not for PE, *F*(1, 95) = 0.64, *p* =.427, η_p_^2^ =.01.

**Fig. 8:**
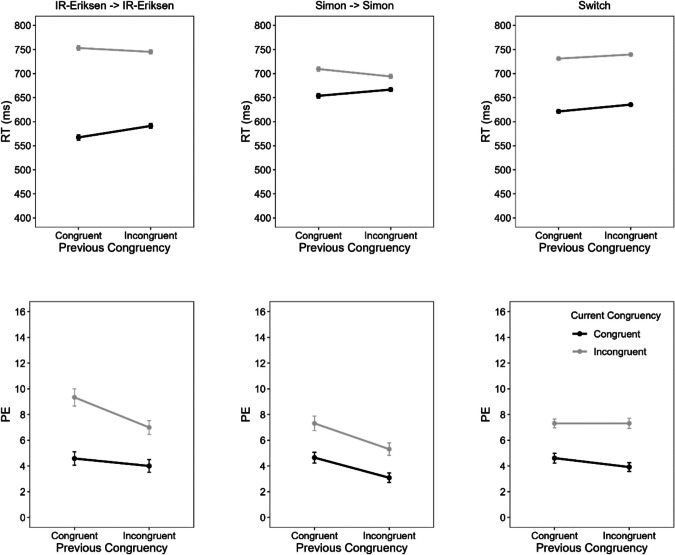
Mean reaction time (RT) and percentage errors (PE) as a function of current congruency (congruent vs. incongruent), previous congruency and task sequence in Experiment 4. Error bars represent ± 1 within-subject *SE* (Morey, 2008)

To ease the comparison with the previous experiments and further explore the four-way interaction, we also conducted ANOVAs separately for each task combination. Specifically, we conducted separate ANOVAs on mean RT and PE with the factors current and previous congruency: (a) focusing only on Simon-task trial repetitions (i.e., CSE between Simon task trials), (b) focusing only on IR-Eriksen trial repetitions (i.e., CSE between IR-Eriksen task trials), and (c) focusing only on trial sequences in which the task changed (i.e., current Simon and previous IR-Eriksen, or current IR-Eriksen and previous Simon) to investigate CSEs between tasks. Figure 8 shows mean RT and PE as a function of current congruency, previous congruency and task sequence.

#### CSE between Simon task trials

For mean RT, there was a significant main effect of current congruency, *F*(1, 95) = 59.78, *p* <.001, η_p_^2^ =.39, and a significant interaction,* F*(1, 95) = 13.56, *p* <.001, η_p_^2^ =.12. Responses were slower in incongruent (702 ms) than in congruent trials (660 ms), and the congruency effect was larger after congruent (56 ms) than after incongruent trials (27 ms), reflecting a CSE of 29 ms. The main effect of previous congruency was not significant, *F* < 1. For PE, both main effects were significant. There were more errors in incongruent (6.3%) than in congruent trials (3.9%), *F*(1, 95) = 21.72, *p* <.001, η_p_^2^ =.19, while there were less errors after incongruent (4.2%) than after congruent trials (6.0%), *F*(1, 95) = 16.81, *p* <.001, η_p_^2^ =.15. The interaction was not significant, *F* < 1.

#### CSE between IR-Eriksen task trials

For mean RT, there was a significant main effect of congruency, *F*(1, 95) = 830.50, *p* <.001, η_p_^2^ =.90, and a significant interaction, *F*(1, 95) = 11.28, *p* =.001, η_p_^2^ =.11. Again, responses were slower in incongruent (749 ms) than in congruent trials (579 ms), and the congruency effect was larger after congruent (186 ms) than after incongruent trials (154 ms), reflecting a CSE of 32 ms. The main effect of previous congruency was not significant, *F*(1, 95) = 3.56, *p* =.062, η_p_^2^ =.04. For PE, there were significant main effects of congruency, *F*(1, 95) = 33.72, *p* <.001, η_p_^2^ =.26, and previous congruency, *F*(1, 95) = 9.44, *p* =.003, η_p_^2^ =.09, but no significant interaction, *F*(1, 95) = 2.90, *p* =.092, η_p_^2^ =.03. Participants made more errors in incongruent (8.2%) than in congruent trials (4.3%), but less errors after incongruent (5.5%) than after congruent trials (7.0%).

#### CSE between Simon and IR-Eriksen tasks

For mean RT, there were significant main effects of current congruency, *F*(1, 95) = 796.53, *p* <.001, η_p_^2^ =.89, and previous congruency, *F*(1, 95) = 19.32, *p* <.001, η_p_^2^ =.17. Responses were slower in incongruent (735 ms) than in congruent trials (628 ms), and also slower after incongruent (688 ms) than after congruent trials (676 ms). Importantly, there was no evidence for a CSE between the two tasks, *F*(1, 95) = 0.89, *p* =.348, η_p_^2^ =.01. For PE, there was only a significant main effect of congruency, *F*(1, 95) = 36.73, *p* <.001, η_p_^2^ =.28, all other *p*s >.165. Participants made more errors in incongruent (7.3%) than in congruent trials (4.3%). We also checked whether the effects depend on whether participants switched from the Simon to the IR-Eriksen task or vice versa by conducting an ANOVA with the factors current congruency, previous congruency, and task sequence (Simon-Eriksen vs. Eriksen-Simon). There were no significant three-way interactions either for mean RT or PE, *F*s < 1 (and the two-way interactions between current and previous congruency were also not significant, *p*s >.374).

### Discussion

The results of Experiment 4 again provide no evidence for domain-general control, as there was no cross-task CSE even when optimizing all conditions to observe a CSE in a confound-minimized paradigm. Again, the results support a domain-specific control account of the CSE, even when the timing of distractor processing is matched between tasks. Importantly, Experiment 4 provided clear evidence for within-task CSEs indicating that a lack of CSE transfer cannot be attributed to the particularities of the confound-minimized design used in Experiments 3 and 4.

#### Additional analyses of Experiments 1–4

The results of Experiments 1–4 indicate that the CSE is highly task-specific. More precisely, we observed only very little evidence of a CSE between the Simon task and the Eriksen task when giving the flanker a temporal head start. However, in all experiments, the delta plot of the IR-Eriksen task was numerically more negative than the delta plot of the Simon task, which was generally quite stable (Experiments. 1, 3, and 4) or also slightly negative (Experiment 2). Although these differences in slopes were numerically small (and not significant in Experiments 2 and 3), they indicate that we were not entirely successful in matching the timing of distractor-to-target activation between tasks. To address this issue, we conducted additional analyses (not preregistered). Specifically, for each experiment we calculated the difference between the IR-Eriksen and Simon delta plot slopes for each participant and then split participants into two groups based on the resulting values – that is, participants with small versus large differences in delta plot slopes. We then reanalyzed all results, focusing only on participants with small differences. Note that when comparing the slopes via paired *t*-tests for participants in this small-difference group, there was no significant difference in either experiment (all *p*s >.197). Critically, even for participants in this group, the results remained the same. That is, there were no significant cross-task CSEs (all *p*s >.340) with the exception of Experiment 2, where the cross-task CSE from the Simon to the IR-Eriksen task was still significant (*p* =.011).

## General discussion

In the present study, we conducted four experiments (with *N* = 373 in total) to investigate whether one hallmark effect of control adaptation, the CSE, can transfer across conflict tasks with different types of distractors (i.e., location in the Simon task and flankers in the Eriksen task). Previous studies generally supported domain-specific accounts of the CSE, suggesting that participants regulate their control specifically for the task-specific conflict created by the distractor (e.g., Egner, 2007; Funes et al., 2010; Lee & Cho, 2013; Wendt et al., 2006). However, since distractors are often processed at different speeds, and therefore conflict type and the dynamic of conflict processing are confounded, the lack of CSE transfer across tasks may also be explained by participants adapting their control based on when conflict occurs (i.e., when distractor processing interferes with target processing) rather than on the specific type of conflict (e.g., perceptual vs. motor conflict).

To test whether cognitive control adjustments are task-specific or can generalize across conflict tasks with different distractors, we investigated the potential transfer of the CSE between a standard Simon task and Eriksen tasks, where flankers were presented before, simultaneously with, or after the target (IR-Eriksen, standard Eriksen, and RI-Eriksen). Since location in the Simon task is typically processed faster than the flankers in the Eriksen task, giving the flankers a head start in processing in the IR-Eriksen task should help to better align the relative timing of distractor-to-target processing between the conflict tasks. Indeed, delta plots, which capture the time-course of distractor-to-target processing, no longer showed the typical increase seen in the Eriksen task. Instead, they remained stable or even partially decreased in the IR-Eriksen task, generally aligning more closely with the temporal characteristic of the Simon task, for which stable (Experiments 1, 3, and 4) or decreasing (Experiment 2) delta plots were observed. Critically, there was little to no evidence of CSE transfer across these two tasks. The only cross-task CSE we observed in Experiment 1 was specific to the task sequence (only from the Simon to the IR-Eriksen task) and could not be replicated in Experiment 2. Furthermore, there were no significant cross-task CSEs in Experiments 3 and 4, and the highly significant same-task CSE in Experiment 4 clearly demonstrates that our paradigm was generally capable of capturing such adaptation effects. Of course, the present study does not rule out the possibility that the cross-task CSEs could be even smaller than those considered with the present sample sizes (see sample size justification in the *Method* section of Experiment 1), and hence may require larger sample sizes. However, considering the rather consistent result pattern across four experiments (*N* = 373 in total),^4^ we suggest the present results do not support the hypothesis that cognitive control adjustments can generalize across different conflict tasks when the temporal dynamics of conflict processing are aligned between the tasks.

Considering that the temporal characteristics of the Eriksen flanker and Simon tasks in previous studies usually markedly differed, as reflected in increasing (Eriksen) versus decreasing (Simon) delta plots, our approach was generally successful in reducing these differences. Nevertheless, in all experiments the delta plots in the Eriksen task were significantly more negative than the (rather stable) delta plot of the Simon task. However, these differences were numerically small, and even when the timing of conflict processing between these tasks was more strongly matched in additional analyses, there was no evidence of a CSE transfer across tasks. Thus, results from the additional analyses provide further evidence in favor of domain-specific over domain-general cognitive control. Nevertheless, the Simon delta plots in the present study were rather stable (in three out of four experiments), rather than being entirely decreasing (e.g., Mittelstädt & Miller, 2020) or showing an inverted U-shaped pattern as in previous studies (e.g., Ulrich et al., 2015). As (at least partially) decreasing delta plots are typically only observed in classic Simon task studies (e.g., with two response keys), the lack of a decrease in Experiments 3 and 4 might reflect that irrelevant location information is generally processed differently (e.g., slower relative to target processing) in more complex, confound-minimized designs (e.g., with four response keys). However, this seems unlikely as Lee and Cho (2024) observed decreasing delta plots for location-based Simon tasks, even though they employed a more complex, confound-minimized paradigm (alternating horizontal and vertical Simon tasks on a trial-by-trial basis) using unimanual aimed-movement responses. Whilst it is possible that different stimulus properties influence the shape of the resulting delta plots (see Gade et al., 2020), as we used a relatively standard Simon-like configuration (horizontal presentation and single-letter stimuli), it seems (more) likely that the non-clearly decreasing Simon delta plots observed here result from the dual-task context (i.e., mixed blocks of Simon and Eriksen task trials).

As mentioned before, several studies have emphasized the importance of considering the timing of distractor-to-target processing to observe a confound-minimized CSE (e.g., Weissman et al., 2014), which is a more direct marker of higher-level cognitive control adaptation than the CSE in designs that do not rule out adaptation based on lower-level memory learning. Specifically, a confound-minimized CSE is only observed when the S-R translation is faster for the distractor than for the target, as is typically the case in the Simon task or in prime-probe or IR-Eriksen tasks where the distractor is given a head start relative to the target (e.g., Weissman et al., 2014). According to the response-modulation account, only under these conditions do control processes have time to adjust the activation of the response triggered by the distractor (e.g., Weissman et al., 2014). Consistent with this account, we observed a confound-minimized CSE for both the Simon and the IR-Eriksen task. Specifically, whereas in Experiments 1–3 the Simon and Eriksen-like conflict tasks alternated on a trial-by-trial basis, in Experiment 4, the task to perform was randomly determined, allowing us to investigate both cross-task and same-task CSEs, with only the latter being significant. Thus, Experiment 4 also demonstrates that the lack of a cross-task CSE in the previous experiments was likely not due to a predictable task sequence. Since there was no evidence of transfer between tasks, the present results provide new support for a domain-specific, rather than a domain-general, response modulation account. Of course, it is possible that the lack of transfer across tasks, even with relatively faster S-R processing, is due to task-specific processing differences. For example, presenting the distractor before the target in the Eriksen flanker task may introduce additional preparatory processes that influence task processing in ways not typically seen when the distractor and target are presented simultaneously (as in the Simon task). Therefore, future studies might explore the potential for distractor-general response modulation by manipulating the speed of different distractors with simultaneous distractor-target presentation (e.g., by varying distractor discriminability; see, e.g., Ellinghaus et al., 2024a; Zeischka et al., 2011).

As the present results suggest that the timing differences between the Simon and Eriksen flanker tasks cannot explain why control operates in a task-specific way, the question arises as to which specific components in conflict processing drive control to produce conflict-task-specific adaptations. Considering that the target sets were identical for both tasks (e.g., H = left response, S = right response), mechanisms related to the distractor type must be considered. For example, it seems possible that in the Eriksen flanker task, the distractors induce more conflict in the perceptual system (stimulus-based conflict) than in the motor system (response-based conflict), whereas the opposite may be true for the Simon task (e.g., Kornblum, 1990; Lavie & Tsal, 1994; Stürmer et al., 2002). In this case, participants may primarily upregulate control during perceptual processing in the Eriksen task (e.g., by biasing spatial attention; see White et al., 2011) but during motor processing in the Simon task when experiencing conflict. That the nature of the distractor type, rather than its timing, is crucial for control adaptations in conflict tasks also aligns with a recent study by Lee and Cho (2024),^5^ in which the CSE was investigated between different Simon tasks involving different spatial distractor codes (e.g., arrow direction and physical location) using aimed-movement responses. For example, they found no significant cross-task CSEs between horizontal arrow-based and vertical word-based Simon tasks, despite rather similar time courses of these Simon effects (i.e., constant and increasing delta plots). Conversely, they found significant cross-task CSEs between horizontal location-based and vertical arrow-based Simon tasks, despite different time courses (i.e., decreasing and increasing delta plots). Thus, they suggested that the CSE between two Simon-like tasks does not depend on the similarity of their delta plots (and hence the time course of distractor processing), but rather on the extent to which the distractor triggers shared spatial codes.

Furthermore, although the conflict tasks in the present study required processing of similar targets, the different distractors might still lead participants to form different task sets, which could constrain transfer between the two conflict tasks. For example, Grant et al. (2020) observed no transfer between tasks using the prime-probe task when distractors and targets were presented visually rather than auditorily, presumably because the different sensory modalities lead to distinct task sets or modality-specific adaptations (see also Lee & Cho, 2023, for discussion). From this perspective, the present results suggest that different distractor types lead to distinct task sets, even when the task-specific timing of conflict processing is aligned. In future studies, it may be useful to extend the current approach of aligning the temporal dynamics of conflict processing across two tasks – each with a unique distractor type – to a single task that incorporates two distractor types (see Egner, 2007; Schlaghecken & Maylor, 2020). For example, presenting a target flanked by distractors on either the left or the right side (e.g., Rey-Mermet et al., 2021) allows researchers to investigate how the two distractor types influence conflict processing within a trial (“within-trial CSE”) and how participants adapt their conflict processing in the following trial based on distractor-specific congruency (“between-trial CSE”). A study by Schlaghecken and Maylor (2020), using a confound-minimized hybrid prime-Simon task, suggests that in such designs, control still acts in a domain-specific way, as there was no between-trial CSE, and the observed within-trial CSE reflected a reluctance to reactivate a just-discarded response rather than domain-general control. It remains to be seen whether the same holds true for a hybrid Eriksen flanker-Simon task when the timing of distractor processing is aligned.^6^ Moreover, combining distractor types within a single trial could help to examine how dual-route models like DMC, which guided the present research, might be extended to conceptualize two distractor types within one trial – potentially by assigning separate distractor functions to each.

## Conclusion

In the present study, we demonstrate that the lack of a cross-task CSE between the Simon and Eriksen flanker tasks is unlikely to be explained by differences in the temporal dynamics of conflict processing (i.e., the temporal overlap of target and distractor processing). Despite aligning the temporal overlap of target and distractor processing across the two tasks by presenting the target in the Eriksen flanker task before the distractors, the results showed little, if any, evidence of CSE transfer across the two tasks. These findings provide new evidence for domain-specific rather than domain-general cognitive control, even when the temporal dynamics of conflict processing are controlled for.

## Data Availability

Raw data and preregistrations are available via OSF at https://osf.io/937ba/.

## Footnotes

1 We also focused on the Eriksen flanker task instead of the Stroop task, for which increasing delta plots are typically also observed (e.g., Ellinghaus et al., 2024b; Pratte et al., 2010), for several other reasons. First, whereas both Eriksen and Simon task trials produce only perceptual-related and motor-related informational conflict (see Ellinghaus et al., 2024a; Gratton et al., 1992; Kornblum et al., 1990), the Stroop task introduces additional higher-level task conflict (i.e., between word reading and color naming, e.g., Goldfarb & Henik, 2007; Steinhauser & Hübner, 2009). Second, whereas the Eriksen and Simon tasks are often sensitive to the same experimental manipulations, the Stroop task is not (e.g., reward, Mittelstädt et al., 2023). Thus, the Stroop task appears somewhat special, and it seemed more likely that control can transfer across the more similar Eriksen and Simon tasks when controlling for the timing of target and distractor processing. Third, whereas for both the Eriksen and arrow-Simon tasks the increasing delta plots became more negative when irrelevant information is given a temporal head start in the study by Mackenzie et al. (2022), the Stroop task was largely unaffected by the same or even stronger temporal manipulation.

2 As can be seen in our preregistration, in this and the other experiments, we did not provide a sample size justification. The sample size was determined considering both practical constraints (e.g., participant availability) and empirical constraints (e.g., effect sizes in previous studies, greater variability in an online setting). With the chosen sample size of 100 participants per experiment, we would achieve over 90% power to detect modulations of congruency as a function of previous congruency of at least η_p_^2^ =.10 at a significance level of α =.05. We considered this a reasonable minimum effect size of interest, especially given that same-task CSEs are typically large (e.g., η_p_^2^ >.62 in Schmidt & Weissman, 2014), and previously reported cross-task CSEs, when present, were also relatively large (e.g., η_p_^2^ >.17 in Freitas et al., 2007).

3 Contrary to our preregistration, we do not report separate ANOVAs for the two tasks, as in the overall ANOVA all critical interactions were not significant.

4 In an additional exploratory (not preregistered) analyses, we combined the data from all experiments for the critical cross-task CSE conditions (i.e., using only the IR-Eriksen and Simon blocks in Experiments 1 and 2, and task-switch trials in Experiment 4), and analyzed the current congruency effect as a function of previous congruency when the conflict task switched (ignoring the direction of the switch). However, even in this high-powered analysis, there was no evidence of a significant cross-task CSE, *F*(1, 372) = 1.52, *p* =.218.

5 When the current series of experiments was conceptualized and conducted, the study by Lee and Cho (2024) was not yet published.

6 Note that there are some methodological constraints to consider when attempting to combine an Eriksen task with a flanker head-start and a Simon task within a single trial. Specifically, if flankers are presented before the target and positioned to the left or right, they may already trigger location-based activation. As a result, this approach would likely fail to achieve the intended alignment of the temporal processing of flanker- and location-based conflicts. Therefore, combining a delayed-flanker paradigm with a Simon task requires a carefully designed setup that prevents the flankers from prematurely eliciting location-based activation.

## Code availability

The JavaScript code used to run the experiment and the code used to analyze the data are available upon request.
